# Supplementary material for: Assessing the Pre-Vaccination Anti-SARS-CoV-2 IgG Seroprevalence among Residents and Staff in Nursing Home in Niigata, Japan, November 2020
Source: Viruses. 2022 Nov 21;14(11):2581. doi: 10.3390/v14112581 (PMC9698805; doi:10.3390/v14112581)
Supplement: Supplementary file 1 [file viruses-14-02581-s001.zip › viruses-2037989-supplementary.pdf]

Supplementary Files for: Assessing the Pre-Vaccination Anti-SARS-CoV-2 IgG Seroprevalence among Residents and Staff in Nursing Home in Niigata, Japan, November 2020

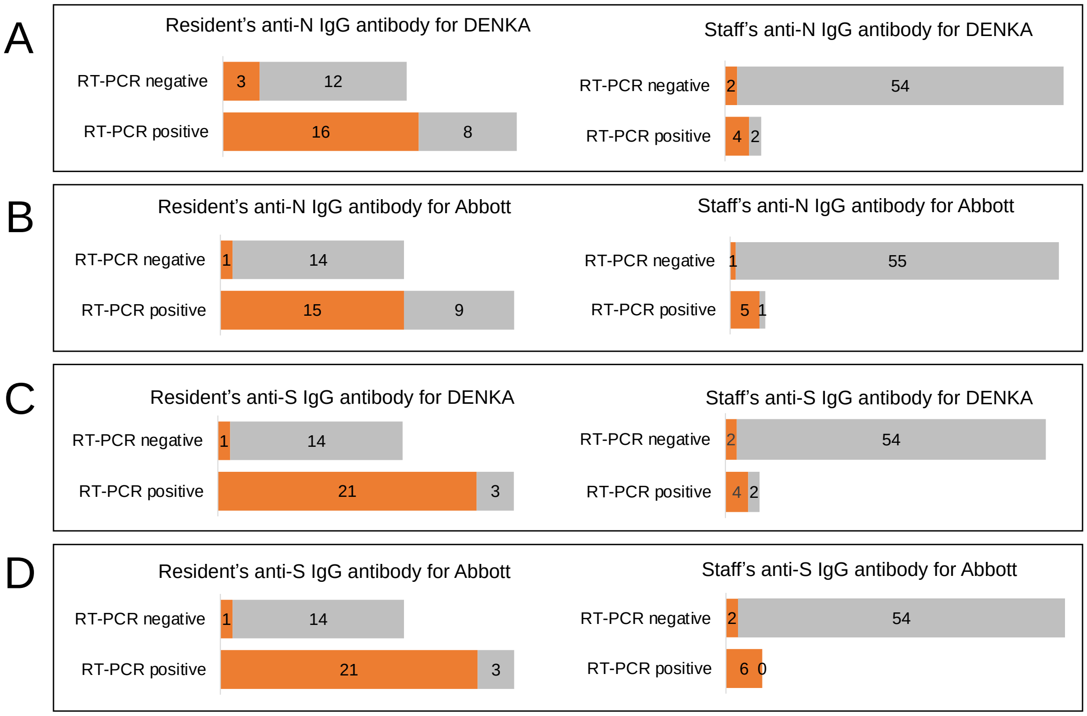

Figure S1. Seroprevalence in RT-PCR-positive and RT-PCR-negative participants (N=101). (**A**) Seroprevalence in reverse transcription PCR (RT-PCR)-positive and RT-PCR-negative participants in resident's and staff's anti-nucleocapsid (N) immunoglobulin G (IgG) antibody for DENKA (Tokyo, Japan). (**B**) Seroprevalence in RT-PCR-positive and RT-PCR-negative participants in resident's and staff's anti-N IgG antibody for Abbott (Illinois, USA). (**C**) Seroprevalence in RT-PCR-positive and RT-PCR-negative participants in resident's and staff's anti-spike (S) IgG antibody for DENKA. (**D**) Seroprevalence in RT-PCR-positive and RT-PCR-negative participants in resident's and staff's anti-S IgG antibody for Abbott. The bars represent the number of seropositive (orange) and seronegative (grey) participants, according to the RT-PCR test results, stratified for staff and residents for both DENKA and Abbott. The numbers in the bars represent absolute numbers.

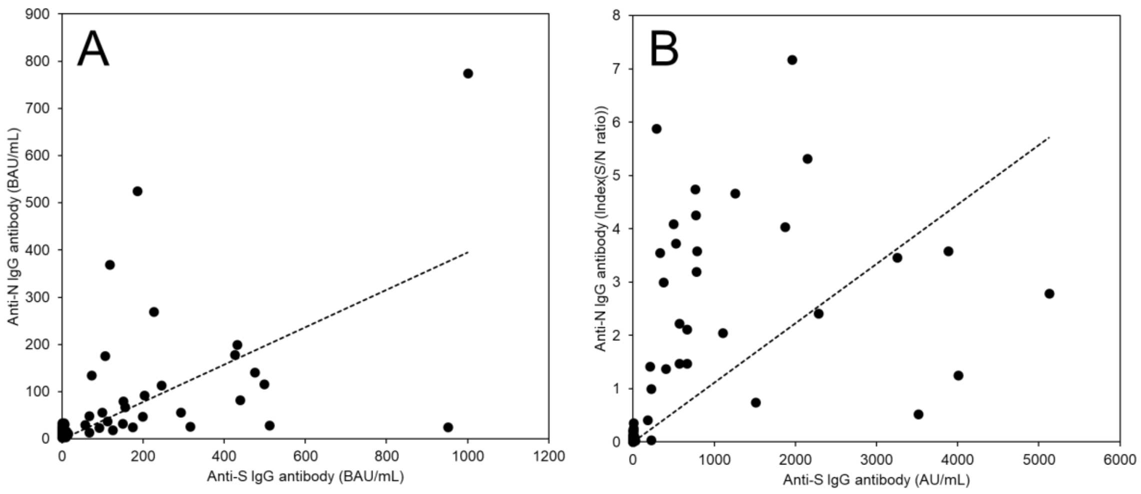

Figure S2. Bivariate relationship between anti-S IgG antibody and anti-N IgG antibody for DENKA and Abbott methods (N=101). Spearman's rank-order correlation coefficients (ρ) between the anti-spike (S) immunoglobulin G (IgG) antibody and anti-nucleocapsid (N) IgG antibody for DENKA (Tokyo, Japan) and Abbott (Illinois, USA) were explored. Results with (**A**) DENKA (ρ=0.64, P<0.01) and (**B**) Abbott (ρ=0.66, P<0.01) are shown. Two samples were excluded because the test could not be performed, and the data analysis was performed on 101 cases.

Table S1. Concordance assessment between DENKA and Abbott methods for anti-N and anti-S IgG antibodies by resident or staff utilizing Cohen's kappa statistic (N=101).

Staff	Cohen's kappa statistic (κ)	95% CI
Anti-N IgG antibody	0.81	0.56 to 1.00
Anti-S IgG antibody	0.83	0.62 to 1.00
**Resident**	**Cohen's kappa statistic (κ)**	**95% CI**
Anti-N IgG antibody	0.63	0.39 to 0.88
Anti-S IgG antibody	0.84	0.68 to 1.00

Abbreviations: CI, confidence interval; N, nucleocapsid; S, spike; IgG, immunoglobulin G.
